# Gut microbiota characteristics and therapeutic effects of fecal microbiota transplantation in children with autism spectrum disorder in central China: a combined cross-sectional and prospective study

**DOI:** 10.3389/fped.2025.1648471

**Published:** 2025-09-02

**Authors:** Rui Wu, Xu Teng, Yunkai Guo, Yongxi Cai, Yongling Lv, Heyun Gao, Wen Zhang, Hexiao Shen, Jingyi Fan

**Affiliations:** ^1^ Department of Pediatric, Zhongnan Hospital of Wuhan University, Wuhan, China; ^2^ Autism Clinical Research Center, Zhongnan Hospital of Wuhan University, Wuhan, China; ^3^ Department of Pediatric Surgery, Zhongnan Hospital of Wuhan University, Wuhan, China; ^4^ School of Life Science, Hubei University, Wuhan, Hubei, China

**Keywords:** fecal microbiota transplantation, autism spectrum disorder, therapeutic effect, intestinal health, gut-brain axis

## Abstract

**Introduction:**

Gut microbiota dysbiosis is implicated in autism spectrum disorder (ASD), yet scalable therapeutic interventions remain limited. This study investigated gut dysbiosis profiles in children with ASD and evaluated the clinical efficacy of a simplified fecal microbiota transplantation (FMT) protocol using pediatric donors.

**Methods:**

In a cross-sectional phase, 48 children with ASD and 51 age-/sex-matched healthy controls underwent gut microbiota analysis. Subsequently, 25 ASD participants received FMT via a streamlined protocol: 3-day bowel preparation followed by 6-day transcolonoscopic microbiota infusion from pediatric donors. Clinical outcomes and microbiota shifts were assessed at 3-month follow-up.

**Results:**

(1) Baseline Dysbiosis: ASD subjects exhibited reduced microbial diversity, with decreased *Faecalibacterium* and *Bifidobacterium* but elevated Megamonas and *Akkermansia* vs. controls. (2) Clinical Efficacy: Post-FMT, significant improvements occurred in core ASD symptoms and gastrointestinal comorbidities. (3) Microbiota Shifts: FMT recipients showed increased beneficial genera (*Prevotella, Faecalibacterium, Agathobacter, Dorea*) and reduced *Escherichia-Shigella*.

**Discussion:**

A simplified pediatric donor FMT protocol effectively modulates gut microbiota composition and alleviates both behavioral and gastrointestinal symptoms in children with ASD. This strategy demonstrates feasibility for clinical translation, highlighting microbiota-targeted therapy as a promising intervention for ASD.

## Introduction

Autism spectrum disorder (ASD) is a complex neurodevelopmental condition that originates in infancy and early childhood. Its core characteristics include persistent deficits in social communication and interaction, as well as restricted interests and repetitive patterns of behavior (1).In recent years, the prevalence of ASD has shown a consistent upward trend. According to data from the Centers for Disease Control and Prevention (CDC), the prevalence of ASD among 8-year-old children rose from 2.27% in 2018 to 2.7% in 2023 (2, 3). National epidemiological surveys in China similarly reported a prevalence of 0.7% among school-aged children (ages 6–12) in 2020 (4), whereas young children (0–6 years) showed a prevalence of 1.8% in 2023 (5). Conservatively estimated, approximately 2 million children aged 0–14 years are currently affected by ASD in China, with 160,000 new cases emerging annually (6). The high disability rate and incurability of ASD have created a significant public health burden.

The pathogenesis of ASD remains incompletely elucidated. Current evidence suggests that its etiology involves an interplay of genetic susceptibility, immune dysregulation, and environmental factors (7).At present, primary interventions predominantly focus on behavioral therapy and educational support, with a significant lack of specific agents aimed at addressing core symptoms (8, 9). Significantly, children with ASD exhibit higher susceptibility to comorbidities-including functional gastrointestinal disorders (e.g., abdominal pain, constipation, diarrhea), sleep disturbances, and emotional/behavioral problems-compared to neurotypically developing (TD) children (10–12). These comorbidities not only substantially impair quality of life but may also exacerbate core behavioral symptoms (13, 14). Consequently, investigating the underlying pathophysiological mechanisms and developing novel interventions are key priorities in research on neurodevelopmental disorders.

In recent years, advances in the gut-brain axis (GBA) theory have intensified investigations into the mechanistic roles of gut microbiota in neurodevelopment. This theory highlights the bidirectional communication between gut microbiota and the central nervous system through multiple pathways including chemical, neural, immune, and endocrine routes, which ultimately modulate brain development and function (15–17). The pathological significance of this axis is now widely recognized, as mounting evidence implicates intestinal dysbiosis as a crucial susceptibility factor in the progression of numerous neurological disorders, including Alzheimer's disease, Parkinson's disease, multiple sclerosis, and notably, ASD (18). In the context of ASD specifically, a consistent body of evidence points to characteristic dysbiosis. The main manifestations are featuring reduced microbial diversity, depletion of beneficial bacteria [including short-chain fatty acid (SCFA)-producing taxa], and enrichment of potential pathogens such as the *Clostridium* genus (19–27). In addition, metabolomic investigations further demonstrate microbial involvement in ASD pathogenesis via dysregulation of key metabolic pathways, including SCFA production, tryptophan metabolism, and phenolic compound synthesis (25, 28–35). Crucially, this dysbiosis is now understood to be a potent driver of immune dysregulation, a core pathological feature in ASD. Emerging studies highlight how an imbalanced gut microbiome can trigger pro-inflammatory responses and compromise intestinal barrier integrity, contributing to the neuroinflammatory states observed in the disorder (36). However, certain heterogeneity persists across studies due to variations in geographic distribution, dietary patterns, antibiotic exposure history, and subtypes of ASD among the cohorts (20, 37–39). Despite this variability, the compelling link between the gut microbiome and ASD pathophysiology has spurred the exploration of microbiota-targeted therapies. This approach is built upon the growing success of such interventions in managing other conditions rooted in microbial dysbiosis. A recent comprehensive review, for example, highlights the therapeutic potential of modulating the gut microbiome for a range of gastrointestinal disorders, thereby establishing a strong rationale for exploring these strategies in neurodevelopmental conditions that frequently present with GI comorbidities (40).

FMT is an emerging intervention that restores intestinal microbial balance and has demonstrated potential in autism spectrum disorder (ASD) management. Preliminary evidence indicates FMT ameliorates gastrointestinal symptoms and partially improves core behavioral manifestations in ASD. In an open-label trial administering microbiota transfer therapy (MTT) to 18 children with ASD for a period of 10 weeks, over 80% experienced relief from gastrointestinal symptoms such as abdominal distension and diarrhea, and the symptom improvement could last for up to 2 years (41, 42). Furthermore, a recent study reported 20% and 17% reductions in Autism Behavior Checklist (ABC) and Social Responsiveness Scale (SRS) scores, respectively, among 40 children following a 4-week FMT protocol (43). Although FMT has shown preliminary therapeutic efficacy in treating ASD, its clinical application encounters significant challenges. Specifically, the lack of standardized protocols for the preparation of fecal bacterial liquid, administration routes and treatment plans has hindered the wide implementation of this treatment measure (44). More importantly, most current studies utilize microbiota from adult donors. However, the developing gut ecosystem in children may display age-related incompatibilities in microbial composition and metabolic functions when compared to adult-derived communities. This discrepancy could potentially lead to immune dysregulation and disorders related to sexual development (45, 46). Currently, there is still a lack of a systematically constructed child-specific donor microbiota bank, which has become one of the main limiting factors for the application of FMT in the pediatric ASD population. Therefore, exploring the pediatric donor microbiota with clearly defined sources and age-matched is anticipated to offer a more suitable microecological foundation for the safety and efficacy of FMT in pediatric ASD.

In light of the aforementioned context, this research is structured into two distinct components. The first component consists of a cross-sectional cohort study designed to systematically assess the differences in intestinal microbiota characteristics between children diagnosed with ASD and healthy control children in the central region of China. The second component involves a single-center, self-controlled open-label study that will administer FMT via colonoscopy, utilizing microbiota from pediatric donors. This intervention seeks to evaluate the relationship between clinical improvements and behavioral symptomatology in children with ASD, both prior to and following the treatment. Collectively, this study aims to establish a foundational understanding of the mechanisms underlying FMT, enhance the development of individualized treatment strategies, and establish a safety framework for the application of FMT in pediatric populations.

## Materials and methods

### Research design

This research design is divided into two parts: (1) Cross-sectional cohort study: We recruited 48 children with autism spectrum disorder (ASD) aged 3–17 years meeting the Diagnostic and Statistical Manual of Mental Disorders (5th ed.; DSM-5) (1) criteria, along with 51 age/sex-matched healthy controls, were recruited. To analyze gut microbiota structural profiles in ASD. (2) Single-center, self-controlled open-label trial: Among the 48 ASD participants, 25 eligible patients were selected through physician screening to receive fecal microbiota transplantation (FMT). Changes in ASD core symptoms and gut microbial composition were assessed using standardized rating scales and fecal microbial analyses pre- and post-intervention to investigate therapeutic effects of FMT. This study was approved by the Medical Ethics Committee of Zhongnan Hospital, Wuhan University in accordance with the Declaration of Helsinki. Before enrolling in the study, written informed consent was obtained from all participants or their legal guardians. Furthermore, the study was registered at the Chinese Clinical Trial Registry (www.chictr.org.cn). The trial protocol is accessible through the registration number ChiCTR2500105006.

### Recruitment and grouping of subjects

ASD group: From December 2023 to January 2025, patients with ASD who met the diagnostic criteria of DSM-5 were recruited from the pediatric outpatient department of Zhongnan Hospital of Wuhan University.

Healthy Control (HC) Group: Healthy children and adolescents without neurodevelopmental disorder histories were recruited from public kindergartens and schools in Wuhan.

Enrollment Criteria for the FMT treatment group: (1) Aged 4–17 years old; (2) ASD diagnosis confirmed by at least two experienced child neurologists per DSM-5 criteria; (3) Legal guardians fully understand the research protocol and sign a written informed consent form.

Exclusion criteria: (1) Antibiotic or probiotic use within 3 months prior; (2) Coagulation disorders; (3) Severe gastrointestinal diseases (e.g., intestinal obstruction) or major organic pathologies; (4) Presence of infectious diseases; (5) Presence of other mental disorders.

### Donor screening and FMT treatment for the ASD patients

FMT donor preparation was exclusively conducted by Maintainbiotech. Ltd. (Wuhan), which has been pre-approved by the hospital. Donor screening strictly adhered to the Chinese FMT Donor Screening Guidelines (47). Specifically, donors were selected from healthy control (HC) volunteers meeting health criteria in Part 1 of this study, and were further screened and evaluated according to guideline to ensure microbial safety and suitability. Written informed consent was obtained from guardians for the use of donor samples. Fecal microbiota suspension preparation rigorously followed the Chinese FMT Preparation Guidelines (48): Donor feces were collected in sterile containers, and 200 g of feces were immediately mixed uniformly with 500 ml of 0.9% sterile saline. The resulting suspension was filtered through sterile gauze to remove large particulate matter. The suspension was centrifuged at 3,000 rpm for 10 min at 4 ℃ to remove precipitates. The resulting bacterial suspension was stored at −80 ℃ and resuscitated in a 37 ℃ water bath before use.

Three days prior to FMT, subjects initiated a liquid diet and bowel preparation with oral rifaximin (0.1 g three times daily) plus polyethylene glycol 4,000 powder. Bowel preparation adequacy was confirmed when colorless or light yellow transparent watery stools were discharged. On transplantation day, a catheter was inserted into the ileocecal region via colonoscopy. Fifty milliliters of fecal suspension, rewarmed to 37 ℃ in a water bath, were infused through the catheter. Transplantations were performed once daily for six consecutive days. Food was restricted within 2 h before and after each infusion, with subjects maintaining a right lateral position for 2 h post-infusion. A liquid diet was sustained throughout FMT. Following treatment completion, gluten-containing foods were strictly avoided for at least 1 month.

### Evaluation and sample collection

Fecal sample collection: Baseline stool samples were obtained from both the ASD and HC groups. The FMT treatment group was resampled pre-intervention and at the 3-month post-treatment time point. All samples were immediately aliquoted and stored at −80 ℃ for subsequent microbiological analyses.

Behavioral and comorbidity assessment: An assessment battery including the Autism Behavior Checklist (ABC) (49), Childhood Autism Rating Scale (CARS) (50), and comorbidity questionnaires (e.g., gastrointestinal symptoms and sleep quality) was administered with the cooperation of their guardians to all FMT recipients pre- and post-treatment.

### DNA extraction, microbial sequencing and bioinformatics analysis

Genomic DNA was extracted from fecal samples using the HiPure Stool DNA Mini Kit (Magen, Guangzhou, China) according to the manufacturer's protocol. DNA concentration was quantified using a Qubit 4 Fluorometer with the Qubit dsDNA HS Assay Kit (Thermo Fisher Scientific, USA), while integrity was assessed via 1% agarose gel electrophoresis. The V3-V4 region of the bacterial 16S rRNA gene was amplified using primers 341F (5’-CCTACGGGNGGCWGCAG-3’) and 805R (5’-GACTACHVGGGTATCTAATCC-3’) in a reaction system containing KAPA HiFi HotStart ReadyMix (Roche, Switzerland), performed on a Veriti Thermal Cycler (Applied Biosystems, USA). PCR amplicons were purified using AMPure XP beads (Beckman Coulter, USA) and subjected to paired-end sequencing (2 × 250 bp) on the Illumina MiSeq platform. Quality control of the raw sequencing data was performed using the DADA2 plugin to remove low-quality bases and chimeric sequences, generating high-quality non-redundant feature sequences (Amplicon Sequence Variants, ASVs). Subsequently, ASVs were taxonomically annotated using the SILVA database (Release 138). Alpha diversity of microbial communities was assessed via Shannon and Simpson indices. Beta diversity analysis employed Bray-Curtis dissimilarity matrices visualized through principal coordinates analysis (PCoA). Statistical significance of community structure differences was assessed using ANOSIM (Analysis of Similarities) and permutational multivariate ANOVA (Adonis). To identify differentially abundant taxa across groups, linear discriminant analysis effect size (LEfSe) was employed, with biological relevance evaluated by LDA scores. Spearman rank correlation analysis was performed to examine associations between ASVs and clinical scale scores including the Autism Behavior Checklist (ABC) and Childhood Autism Rating Scale (CARS).

### Statistical analysis

Statistical analyses were conducted using SPSS 26.0. Continuous variables are presented as medians with interquartile ranges (IQR). Normality testing was performed to guide selection of appropriate statistical methods. Between-group comparisons for continuous variables utilized Mann–Whitney *U* or Wilcoxon signed-rank tests based on data distribution characteristics, while categorical data comparisons employed chi-square tests. Unless otherwise specified, all statistical tests incorporated false discovery rate (FDR) correction to control for false positives in multiple testing. Corrected *p*-values < 0.05 were considered statistically significant.

## Result

### Demographic characteristics

This study included a total of 48 patients with ASD aged 3–17 years and 51 age/sex-matched healthy control (HC) children for comparative analysis of gut microbiota. There were no statistically significant differences in age distribution and sex ratio between the two groups (Table 1).

**Table 1:** Demographic characteristics of study participants and description of the FMT subgroup.

Characteristic	Healthy controls (HC) (*n* = 51)	ASD group (*n* = 48)	*P* value (HC vs. ASD)
Gender (male/female)	46/5	44/4	1.000
FMT subgroup (*n* = 25)	–	23/2	–
Age (years, median [IQR)	7 (5–8)	7 (5–8)	0.600
FMT subgroup (*n* = 25)	–	7 (7–9)	–

### Analysis of α and β diversity of gut microbiota in children with ASD and HC

**Figure 1:**
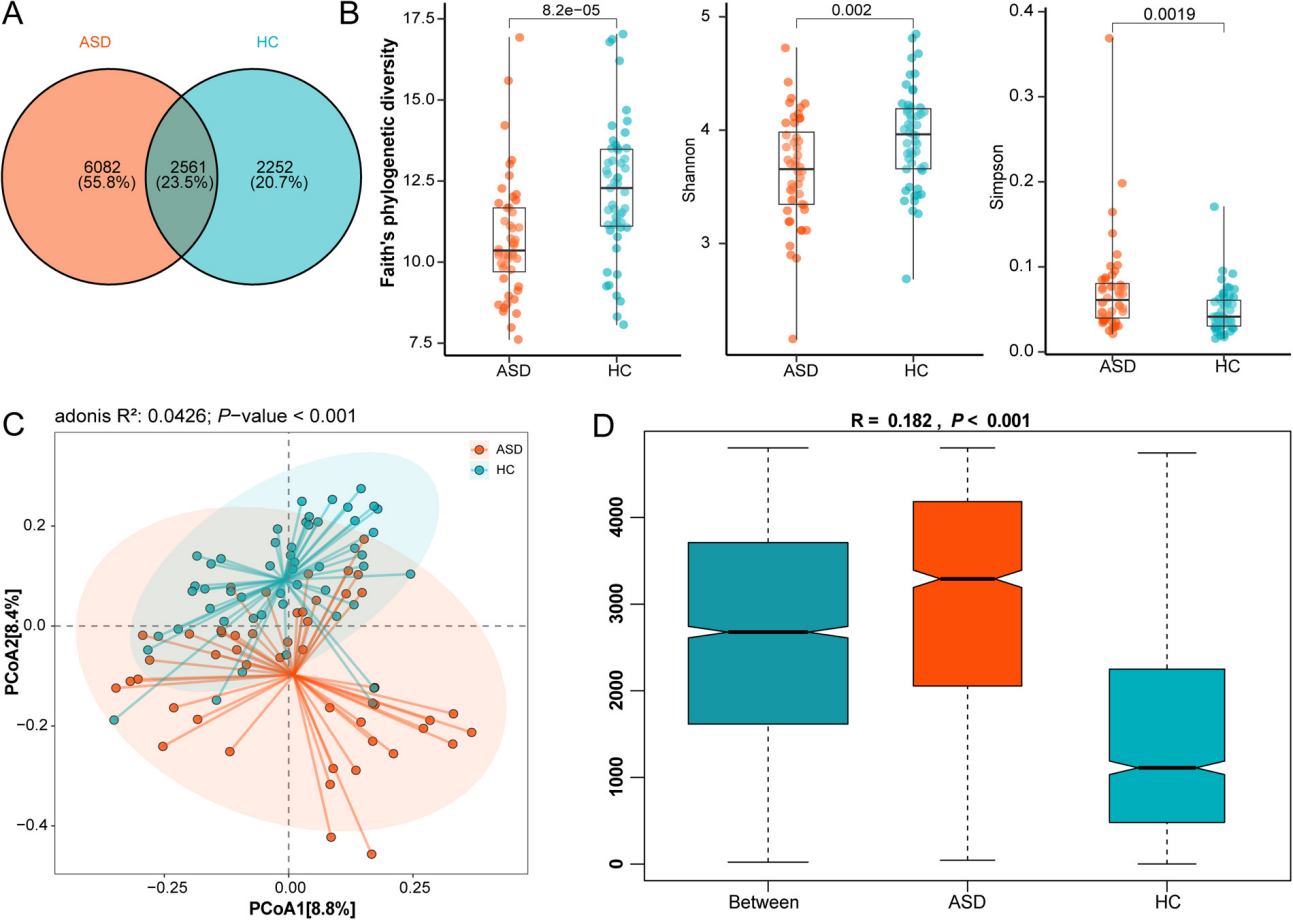
The differences in the composition of gut microbiota between ASD and HC groups. **(A)** The Venn diagram shows the ASV count between the ASD group and the HC group. **(B)** α-diversity indices (Faith's phylogenetic diversity, Shannon, and Simpson) in ASD and HC groups. **(C)** Principal coordinates analysis (PCoA) of β-diversity based on Bray-Curtis dissimilarity between groups, analyzed by Wilcoxon rank-sum test. **(D)** weighted analysis of similarity (ANOSIM) was conducted based on Bray-Curtis distance matrices. The ANOSIM R values indicate inter-group microbial differences, along with their corresponding significance levels (*p* < 0.05).

Venn analysis revealed that only 23.5% of ASVs were shared between the ASD and HC groups (Figure 1A). The proportion of unique microbial communities in the ASD group is significantly higher than that in the HC group (55.8% vs. 20.7%), indicating that the gut microbiome composition of ASD patients has significant specificity. Further analysis revealed significantly decreased Faith's phylogenetic diversity index (*p* < 0.0001) and Shannon index (*p* = 0.002), along with increased Simpson index (*p* = 0.0019; Figure 1B) in the autism spectrum disorder (ASD) group compared to healthy controls (HC). These findings collectively indicate reduced *α*-diversity in the gut microbiota of ASD subjects. The PCoA based on Bray-Curtis distance shows significant separation in the spatial distribution of the two groups of microbial communities (*p* < 0.001, Figures 1C,D), confirming the structural abnormalities of the gut microbial ecosystem in ASD patients at the community level.

### The composition of gut microbiota in children with ASD and HC groups

**Figure 2:**
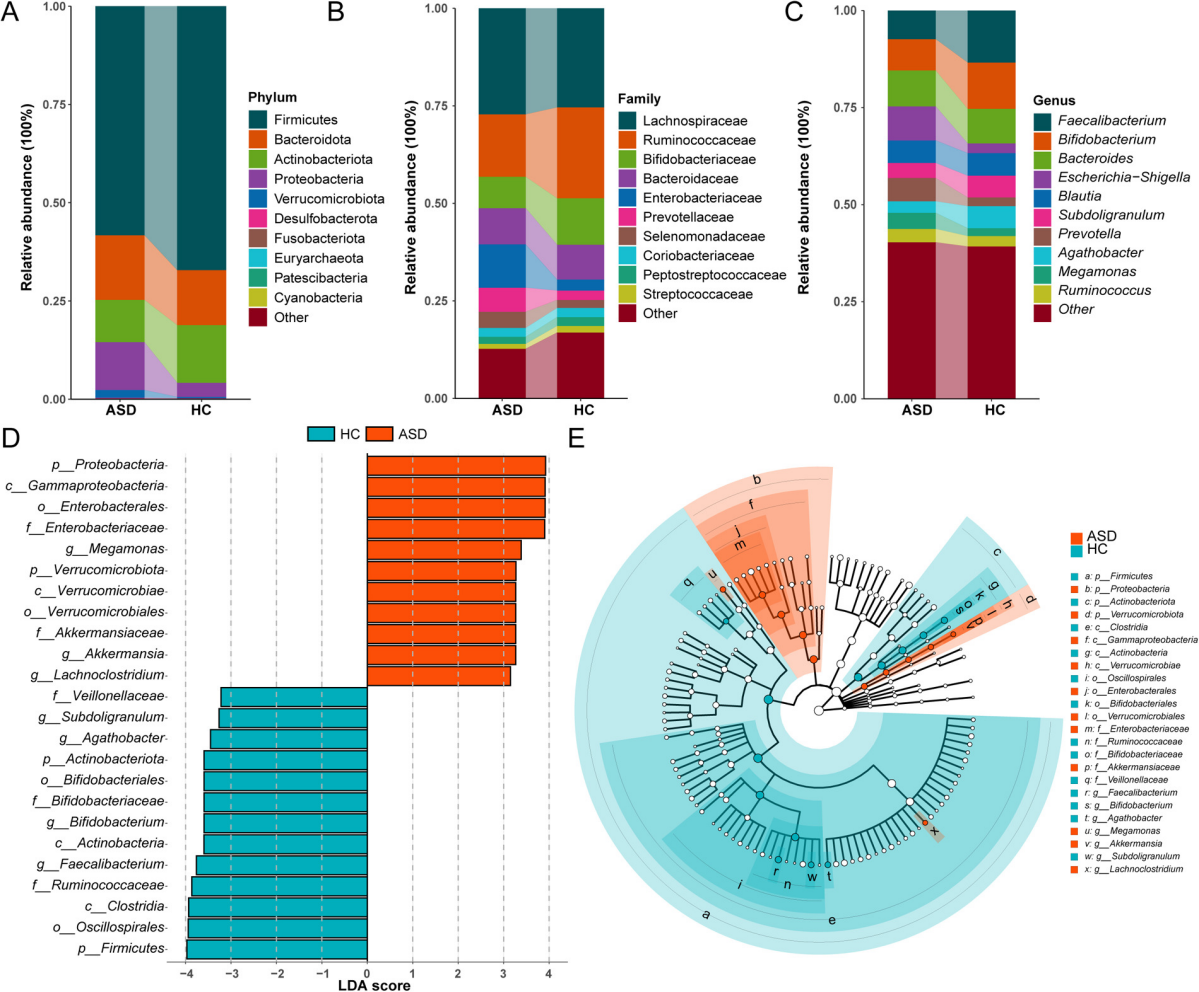
Differences in the composition of gut microbiota between ASD and HC children. **(A)** Phylum level bar chart. **(B)** Family level bar chart. **(C)** Phylum level bar chart. **(D)** Bar graph of LDA scores (LDA > 3). **(E)** Cladogram of the LEfSe analyses.

Significant differences in gut microbiota composition were observed between patients with ASD and HC children. At the phylum level, the ASD group exhibited significantly higher relative abundances of Bacteroidota, Proteobacteria, and Verrucomicrobiota, while showing significantly reduced abundances of Firmicutes and Actinobacteriota compared to the HC group (Figure 2A). Family-level analyses revealed significantly higher relative abundances of Enterobacteriaceae and Prevotellaceae, but lower abundances of Bifidobacteriaceae and Ruminococcaceae in the ASD group compared to HC (Figure 2B). At the genus level, significantly reduced relative abundances of *Faecalibacterium*, *Bifidobacterium*, and *Agathobacter* were observed in the ASD cohort, whereas *Prevotella* and *Escherichia-Shigella* exhibited increased abundances compared to HC (Figure 2C).

LEfSe analysis further identified differentially abundant genera between groups. Significantly increased relative abundances of *Megamonas*, *Akkermansia*, and *Lachnoclostridium* were observed in the ASD group, whereas *Faecalibacterium*, *Bifidobacterium*, and *Subdoligranulum* exhibited reduced abundances in the ASD group (LDA score >3.0; Figures 2D,E).

### The improvement effect of FMT on core symptoms and comorbidities of ASD

**Figure 3:**
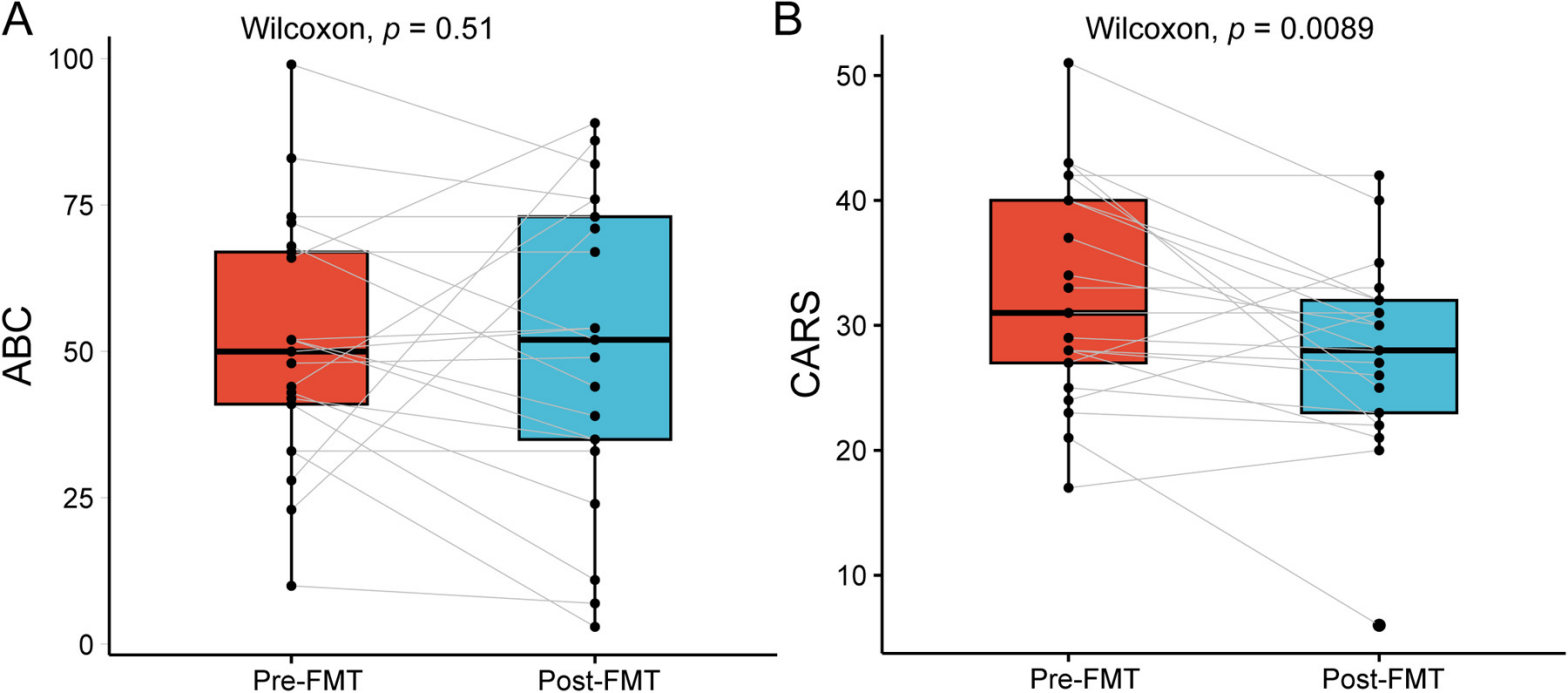
Scores of ABC and CARS scales before and after FMT treatment. **(A)** ABC scores. **(B)** CARS scores. Red: pre-FMT; Blue: post-FMT; The data were tested using Wilcoxon rank-sum test.

Among 25 ASD patients receiving FMT, 21 completed the post-treatment follow-up assessment (4 lost to follow-up due to questionnaire refusal). Assessment scales revealed a 14.6% decrease in mean CARS scores post-treatment vs. baseline (*p* = 0.0089; Figure 3B). Although changes in ABC scores did not reach statistical significance (*p* = 0.51 Figure 3A), the observed downward trend suggests a potential therapeutic effect of FMT on the core symptoms of ASD.

Furthermore, the common comorbidities symptoms of ASD after FMT treatment also showed significant improvement. Pre-treatment, the incidence of comorbidities in these patients was as follows: attention deficit hyperactivity disorder (ADHD, 100%), emotional problems (89.5%), sleep disturbances (73.7%), and gastrointestinal symptoms (52.6%). Post-treatment, gastrointestinal symptoms showed the most pronounced improvement (100% improvement rate). ADHD, emotional problems, and sleep disturbances demonstrated improvement rates of 42.1%, 47.7%, and 42.9%, respectively (Table 2).

**Table 2:** The incidence of common comorbid symptoms in children with ASD before FMT treatment and the improvement rate after treatment.

Symptom	Incidence	Improvement rate
Sleep disturbances	73.7%	42.9%
Difficulty initiating sleep	64.3%	–
Early morning awakening	42.9%	–
Gastrointestinal symptoms	52.6%	100.0%
Constipation	50.0%	–
Emotional problems	89.5%	47.4%
Irritability/aggression	82.4%	–
Hyperarousal	52.9%	–
Attention deficit hyperactivity disorder (ADHD)	100.0%	42.1%

### The impact of FMT on gut microbiota structure in ASD patients

**Figure 4:**
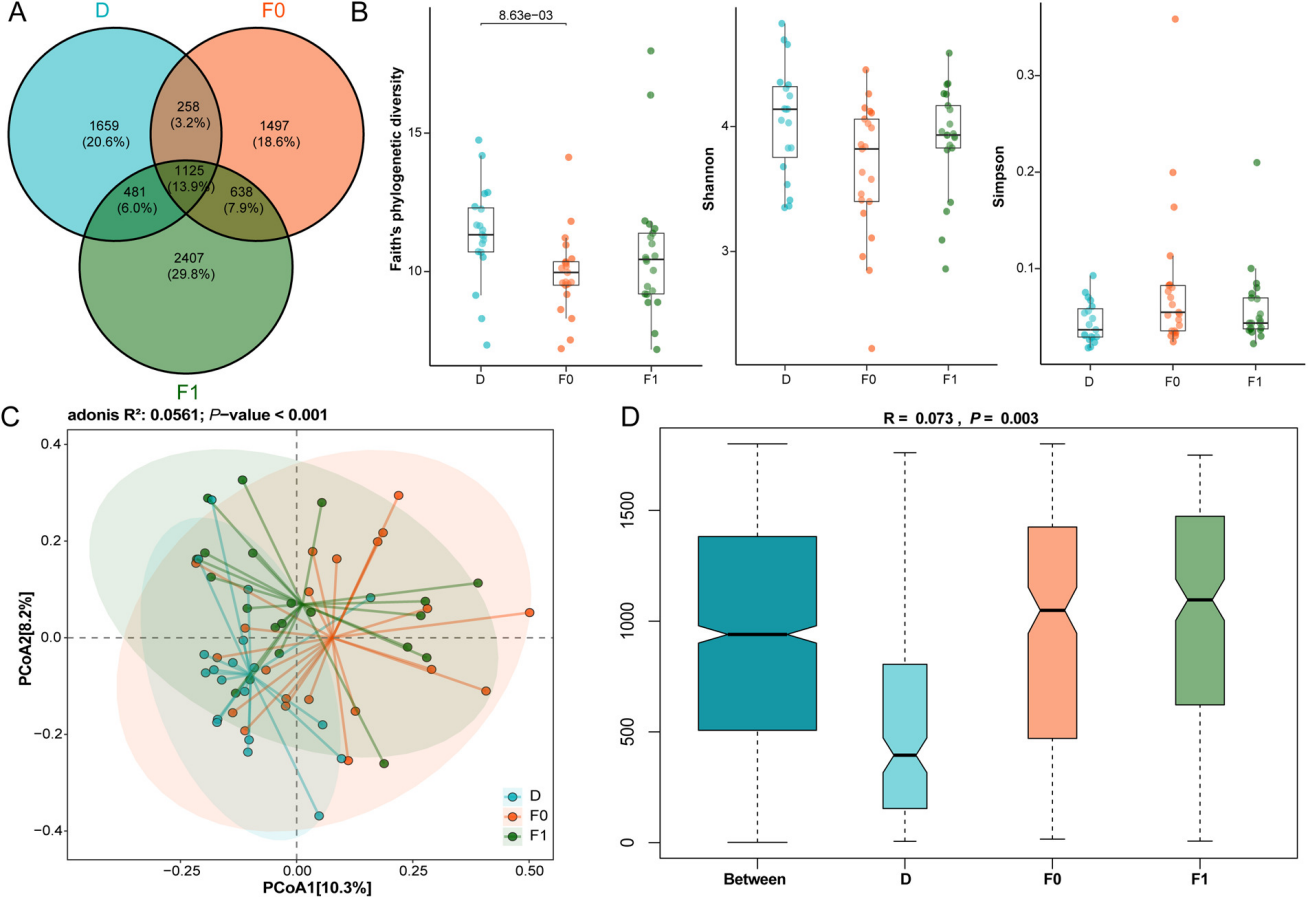
Gut microbiota compositional characteristics before and after FMT treatment. **(A)** Venn diagram illustrates the ASV counts among donors, as well as pre-FMT and post-FMT ASD patients. **(B)** The species α-diversity among groups was assessed by Faith's phylogenetic diversity, Shannon, and Simpson indices. **(C)** PCoA based on Bray-Curtis dissimilarity analyzed β-diversity indices among groups. Data used Wilcoxon rank-sum test. **(D)** Weighted ANOSIMs analysis based on Bray-Curtis dissimilarity distance matrix of fecal microbial communities among three groups. ANOSIM R values showed community differences among three groups with significant *P* values. Blue: Donors **(D)**, Orange: Pre-FMT ASD patients (F0), Green: Post-FMT ASD patients (F1); The data were tested using Wilcoxon rank-sum test.

FMT significantly altered the gut microbiota composition in ASD patients. Venn analysis demonstrated an increase in shared ASV proportions between patients and donors from 17.1% pre-treatment to 19.9% post-treatment (Figure 4A). Although not statistically significant, α-diversity analysis revealed increased diversity post-FMT compared to baseline levels, approaching donor diversity profiles (Figure 4B). PCoA further confirmed significant structural shifts toward donor microbiota in post-treatment samples, indicating effective microbial remodeling by FMT that promoted convergence of the gut microbiota in ASD patients toward healthy donor profiles (Figure 4C).

**Figure 5:**
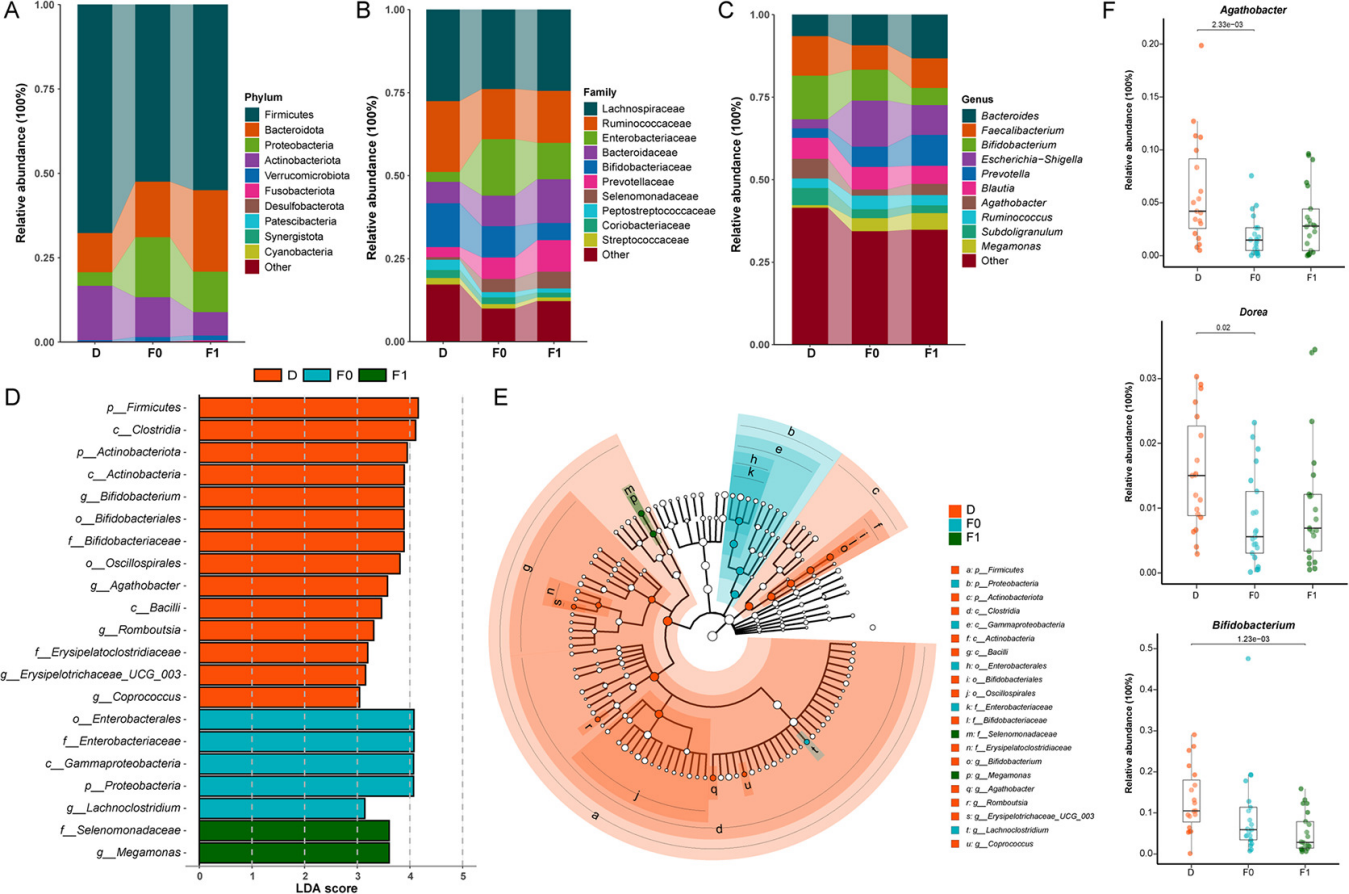
Gut microbiota compositional differences between donors and recipients before/after FMT. **(A)** Phylum level bar chart. **(B)** Family level bar chart. **(C)** Phylum level bar chart. **(D)** Bar graph of LDA scores (LDA > 3). **(E)** Cladogram of the LEfSe analyses. **(F)** The average relative abundance of key bacterial genera.Blue: Donors **(D)**, Orange: Pre-FMT ASD patients (F0), Green: Post-FMT ASD patients (F1); The data were tested using Wilcoxon rank-sum test.

Phylum-level relative abundance analysis revealed increased abundances of Firmicutes and Bacteroidota, but decreased Proteobacteria in the gut of patients post-FMT compared to pre-treatment levels (Figure 5A). At the family level, Ruminococcaceae and Lachnospiraceae exhibited elevated relative abundances, while Enterobacteriaceae showed reduced abundance post-FMT (Figure 5B). At the genus level, increased relative abundances of Prevotella, Faecalibacterium, and Agathobacter were observed alongside significantly reduced Escherichia-Shigella abundance (Figure 5C). Collectively, these shifts demonstrate microbial convergence toward healthy donor profiles.

LEfSe analysis further identified signature taxa with significant inter-group differences: Healthy donors exhibited enrichment of *Bifidobacterium*, *Agathobacter*, and Romboutsia. Following FMT, the predominant genera in ASD patients shifted from *Lachnoclostridium* to *Megamonas* (LDA score >3.0; Figures 5D,E). Analysis of specific genera post-FMT revealed significantly increased relative abundances of *Agathobacter* and Dorea in ASD children, approaching donor levels (Figure 5F). Conversely, the relative abundance of *Bifidobacterium* was lower than pre-treatment baseline levels, contrasting with the common expectation from previous studies that its abundance should increase as a potential beneficial bacterium following effective interventions.

### Correlation analysis between gut microbiota and clinical score

**Figure 6:**
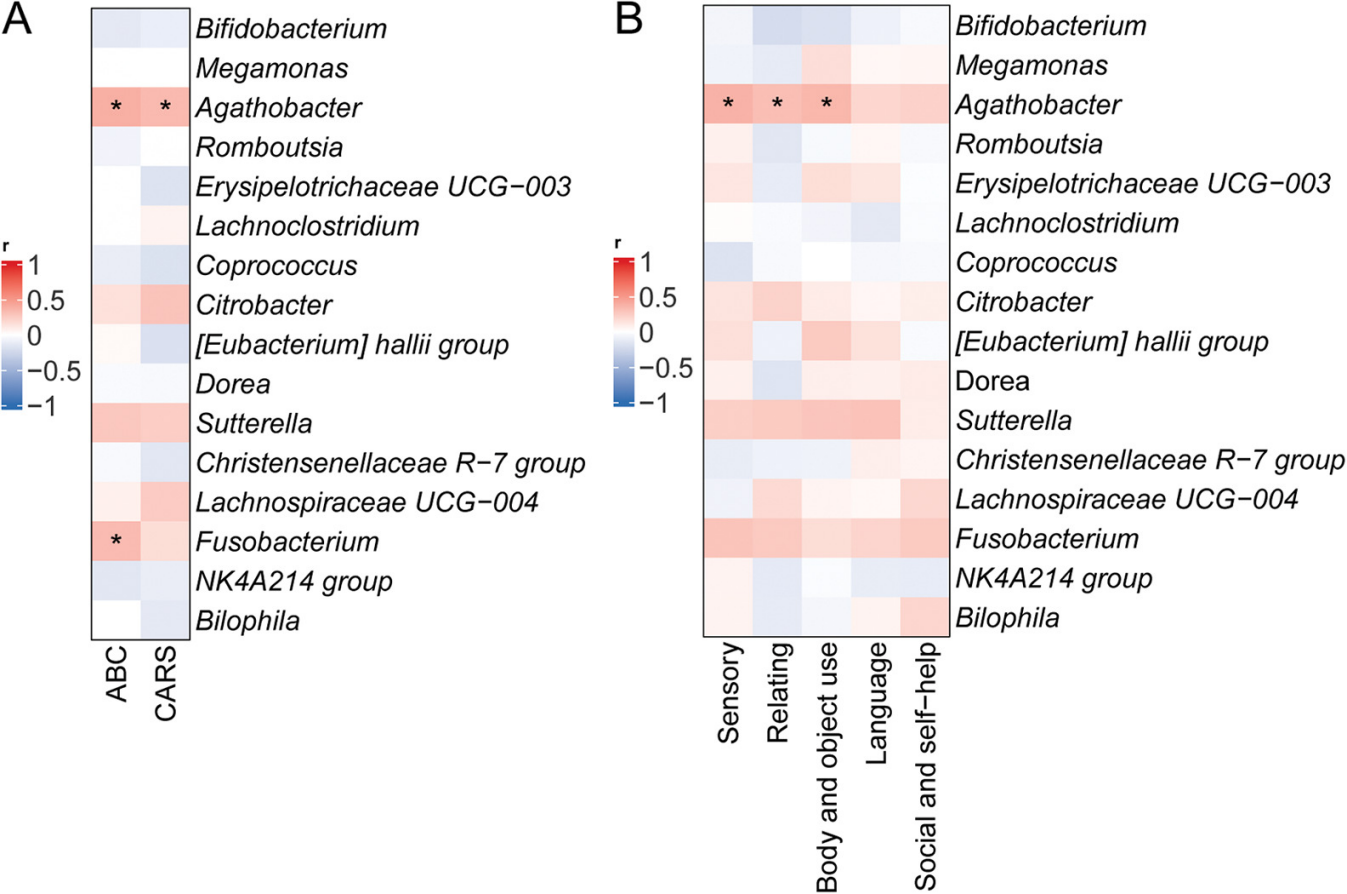
Correlation between relative abundances of specific microbial taxa and ASD symptoms. **(A)** Correlations between relative abundances of specific bacterial genera and total scores on the CARS and ABC. **(B)** Correlations with ABC subscale scores. *p < 0.05.

To investigate relationships between symptoms and specific gut bacterial genera in ASD, correlation analyses were performed between genus-level relative abundances and total scores on the CARS and ABC, including subscale scores (Figure 6). *Agathobacter* relative abundance showed significant positive correlations with both ABC total scores (*r* = 0.56, *p* < 0.05) and CARS total scores (*r* = 0.53, *p* < 0.05), while *Fusobacterium* abundance positively correlated with ABC total scores (*r* = 0.59, *p* < 0.05) (Figure 6A). Analysis of ABC subscale scores further revealed significant positive correlations between *Agathobacter* relative abundance and scores in the Sensory (*r* = 0.49, *p* < 0.05), Relating (*r* = 0.52, *p* < 0.05), and Body/Object Use (*r* = 0.48, *p* < 0.05) domains (Figure 6B).

## Discussion

This study employed a streamlined FMT protocol consisting of three days of bowel preparation followed by six days of transplantation. The intervention resulted in notable improvements in core behavioral symptoms, as evidenced by a 14.6% reduction in CARS scores, and a marked alleviation of gastrointestinal (GI) comorbidities in children with ASD. Interestingly, the changes in the ABC scores did not reach a statistically significance. We hypothesize that the lack of statistical significance could be partly attributed to the limited statistical power resulting from our small sample size. In addition, as a parent-reported checklist, the ABC is inherently subjective, which may introduce variability and affect the sensitivity of the measurement in detecting subtle behavioral changes over the study period. Additionally, significant alterations in gut microbiota composition were observed.

Microbiota diversity analysis revealed distinct dysbiotic features in the ASD group, including a significant reduction in α-diversity and clear separation in β-diversity compared to healthy controls—findings consistent with prior reports by Kang and Ding et al (51, 52). Following the FMT treatment, the microbial profiles of ASD children gradually shifted toward those of healthy donors. This shift was characterized by increased relative abundances of Firmicutes and Bacteroidetes and decreased Proteobacteria at the phylum level. Increased Ruminococcaceae and Lachnospiraceae, and decreased Enterobacteriaceae at the family level. And increased *Prevotella, Faecalibacterium, Agathobacter,* and *Dorea*, with a reduction in *Escherichia-Shigella* at the genus level. These compositional changes were correlated with clinical improvements.

Among these, *Prevotella*—a key genus involved in dietary fiber degradation and vitamin B1 metabolism—showed a positive correlation between its post-FMT increase and improved social behaviors (22). *Faecalibacterium*, particularly *F. prausnitzii*, known for its butyrate production and anti-inflammatory properties, was associated with relief of constipation symptoms, corroborating findings by He et al. (53). The genus *Dorea* may also contribute to maintaining mucosal barrier integrity by modulating Treg/Th17 cell balance (54, 55). Furthermore, the observed reduction in *Escherichia-Shigella* may help ameliorate neurotransmitter imbalances by decreasing aberrant gamma-aminobutyric acid (GABA) synthesis—a phenomenon supported by previous studies demonstrating GABA normalization following FMT (43, 56).

Interestingly, the post-FMT decrease in Bifidobacterium abundance contradicted expectations, as this genus is generally considered beneficial. This unexpected finding may be explained by the “distant attack, close defense” colonization model proposed by Qin et al (57). According to this model, close phylogenetic similarity between donor and recipient strains—such as in this study using pediatric donors and pediatric recipients—may lead to niche competition and colonization resistance, thereby hindering donor strain establishment. This highlights the need for future research to incorporate metagenomic analyses of donor-recipient strain compatibility to optimize FMT outcomes.

The dynamics of Agathobacter abundance revealed a paradoxical pattern. Specifically, its abundance demonstrates a positive correlation with the scores on the ABC and CARS scales. However, following fecal microbiota transplantation (FMT) treatment, there is an observed increase in the abundance of this genus, yet a significant reduction in the scale scores occurs. This apparent contradiction may reflect the genus's complex metabolic capacity. While Agathobacter is a known butyrate producer with anti-inflammatory effects, some strains can also generate metabolites such as succinate, which modulates immunity (58, 59). In the ASD gut environment, succinate may stimulate serotonin (5-HT) production by enterochromaffin cells. Elevated peripheral 5-HT levels have been positively associated with stereotyped behaviors in ASD (60, 61). Furthermore, genetic ablation of intestinal 5-HT synthesis in animal models has been shown to significantly reduce autism-like behaviors (62). Thus, the net clinical effect of Agathobacter may depend on the balance between its beneficial metabolites (e.g., butyrate) and its neuroactive byproducts (e.g., those promoting 5-HT synthesis).

In summary, this study demonstrates that a simplified FMT protocol can effectively alleviate both core behavioral symptoms (CARS scores) and GI comorbidities in children with ASD, with 100% of participants reporting GI symptom improvement. These findings provide preliminary clinical evidence supporting the therapeutic potential of FMT in this population.

However, several limitations must be acknowledged. First, the sample size was relatively small, which may limit the statistical power and generalizability of the findings. Second, the behavioral and GI assessment tools used lacked standardization. Future studies should incorporate internationally validated instruments, such as the Gastrointestinal Symptom Rating Scale (GSRS), Children's Sleep Habits Questionnaire (CSHQ), and Social Responsiveness Scale (SRS), to enhance comparability and objectivity. Third, the study did not include metabolomic analyses, precluding direct validation of gut–brain axis mechanisms through key microbial metabolites such as butyrate and 5-HT. Besides, due to the lack of a double-blind, randomized controlled trial (RCT) makes it difficult to definitively confirm the therapeutic efficacy of FMT and to rule out potential placebo effects. Lastly, although pediatric donors were used, the absence of an adult donor control group limited the ability to directly assess age-specific microbial advantages. Future research should expand sample sizes, integrate metagenomics and metabolomics approaches, and further elucidate microbiota–gut–brain interactions. Randomized controlled trials should also explore the clinical value of age-matched pediatric donor microbiota, thereby laying the groundwork for developing a dedicated pediatric microbiota donor bank.

## Conclusion

This study provides preliminary evidence that a simplified pediatric FMT protocol can improve behavioral symptoms and gastrointestinal comorbidities in children with ASD. The intervention led to measurable changes in gut microbiota composition, some of which were positively correlated with symptom improvement. Key taxa such as *Prevotella, Faecalibacterium*, and *Dorea* may contribute to therapeutic effects via immune modulation, barrier maintenance, and neurotransmitter regulation. The results underscore the potential of age-matched donor microbiota in enhancing colonization compatibility and clinical efficacy. However, larger-scale, multi-omics studies and randomized controlled trials are needed to confirm these findings and inform the development of safe, standardized pediatric FMT protocols.

## Data Availability

The 16S rRNA sequencing datasets presented in the study are deposited in the NCBI SRA repository under accession number PRJNA1273336. Clinical assessment data are available from the corresponding author upon reasonable request, subject to ethical approvals and data protection regulations.
